# Depression patient-derived cortical neurons reveal potential biomarkers for antidepressant response

**DOI:** 10.1038/s41398-021-01319-5

**Published:** 2021-04-01

**Authors:** Yishai Avior, Shiri Ron, Dana Kroitorou, Claudia Albeldas, Vitaly Lerner, Barbara Corneo, Erez Nitzan, Daphna Laifenfeld, Talia Cohen Solal

**Affiliations:** 1 GenetikaPlus Ltd, Givat Ram, Jerusalem, Israel; 2 The Edmond and Lily Safra Center for Brain Sciences, The Hebrew University of Jerusalem, Jerusalem, Israel; 3 Department of Neurobiology, The Alexander Silberman Institute of Life Sciences, The Hebrew University of Jerusalem, Jerusalem, Israel; 4 Columbia Stem Cell Initiative Stem Cell Core, Columbia University Irving Medical Center, New York, NY USA

**Keywords:** Stem cells, Predictive markers, Clinical genetics

## Abstract

Major depressive disorder is highly prevalent worldwide and has been affecting an increasing number of people each year. Current first line antidepressants show merely 37% remission, and physicians are forced to use a trial-and-error approach when choosing a single antidepressant out of dozens of available medications. We sought to identify a method of testing that would provide patient-specific information on whether a patient will respond to a medication using in vitro modeling. Patient-derived lymphoblastoid cell lines from the Sequenced Treatment Alternatives to Relieve Depression study were used to rapidly generate cortical neurons and screen them for bupropion effects, for which the donor patients showed remission or non-remission. We provide evidence for biomarkers specific for bupropion response, including synaptic connectivity and morphology changes as well as specific gene expression alterations. These biomarkers support the concept of personalized antidepressant treatment based on in vitro platforms and could be utilized as predictors to patient response in the clinic.

## Introduction

Major depressive disorder (MDD) is a prevalent mood disorder, affecting over 260 million people worldwide^1^. The World Health Organization declared MDD one of the three leading causes of disability globally, and depressive incidence increased by 14.3% in the last decade^1,2^. There are currently dozens of different antidepressant drugs available^3^, yet only 37% of patients show remission of symptoms after their first line of medication and cumulatively only 56% show remission after the second line of medication^4^. These poor outcomes result from a variety of factors including the unique impacts of medications on each patient’s unique physiology and the significant side effects each patient experiences when being treated with antidepressants.

Several synaptic mechanisms underlying MDD have been suggested to be involved in the patient response to antidepressant treatment, including overall reduced dendritic complexity^5,6^, reduced synaptic content and function^7,8^, and altered metabolism in specific brain regions^9^. Similar mechanisms were also recapitulated by MDD models in vitro, including gene expression alterations, synaptic deficits^10^, and serotonin-receptor hyperactivity^11^. However, due to their complexity, these findings have yet to make their way into clinical realm, forcing physicians to rely on insufficient guiding-tools for treatment selection and giving rise to a largely trial-and-error process for selection. Together with multiple weeks required for treatment response establishment^12^, seeking treatment for MDD often results in months of patient suffering and lost productivity, alongside high healthcare expenses.

Animal models of MDD have been broadly used to shed light on disease mechanisms, despite limited translational ability to aid clinical research^13^. Induced pluripotent stem cells (iPSCs) present an invaluable platform for disease modeling and drug discovery, as they maintain patient genetics and are capable of differentiating into any cell type^14^. Reprogramming of patient cells to iPSCs and further differentiating them toward neurons has already been shown to be beneficial for modeling several neuropsychiatric diseases including bipolar disorder^15^, schizophrenia^16,17^, and MDD^11,18^. In all these models, differentiated neurons derived from patient cells revealed relevant molecular and cellular features compared with healthy control cells. Specifically, one study identified baseline changes in synaptic density and neuronal function using calcium imaging between neurons derived from iPSCs of healthy subjects and of patients with mental disorders in vitro^10^. These changes corresponded to features identified using animal models and human postmortem or brain imaging studies, suggesting they encompass relevant aberrant physiological features.

However, most of these studies used fibroblasts as iPSC sources, which are relatively hard to collect from patients and often bear mutations that could affect their derivatives^19^. Furthermore, most cortical neuron differentiation protocols require months for neural maturation, preventing them from becoming a timely alternative for trail-and-error drug prescribing approach^11,20^.

Lymphoblastoid cell lines (LCLs), derived from patient cells, have been suggested as a replacement of fibroblasts as iPSC source. The Sequenced Treatment Alternatives to Relieve Depression (STAR*D) is a study initiated by the National Institute of Mental Health (NIMH), aimed to improve the understanding of treatment options in MDD patients. The study included over 4000 patients diagnosed with MDD, who were all treated with citalopram, a commonly prescribed selective serotonin reuptake inhibitor (SSRI)^21,22^. Upon symptom persistence, up to four other treatments were offered. Patients were followed for up to 12 months, using several psychological evaluation scales. Blood samples drawn at baseline enable retrospective research into the biology of MDD and patient-specific drug response^21,22^.

**Fig. 1:**
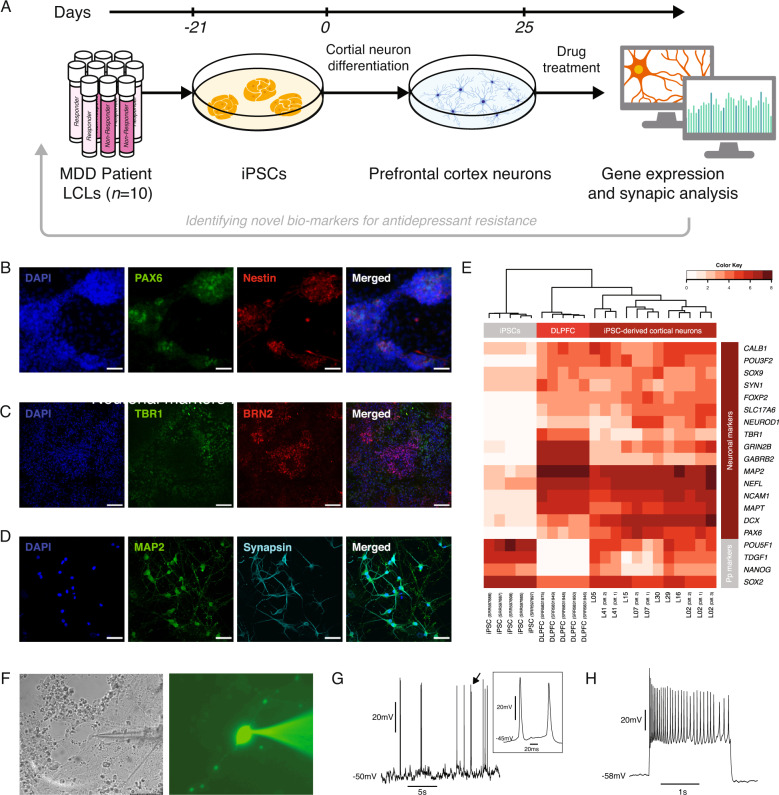
MDD patient LCLs reprogrammed to iPSCs and differentiated into functional cortical neurons. **A** Schematic illustration protocol and analysis. Major depression disorder (MDD) patient lymphoblastoid cell lines (LCLs) were reprogrammed into induced pluripotent stem cells (iPSCs) using Sendai viruses. iPSCs were then rapidly differentiated into cortical neurons, treated with an antidepressant and analyzed thereafter using immunofluorescent staining and RNA-sequencing. **B** Five days into the differentiation protocol, patient-derived cells were positive for the neural progenitor markers PAX6 and Nestin. Scale bars represent 260 µm. **C** After 25 days, neurons were positive for the cortical neural markers TBR1 and BRN2. Scale bars represent 100 µm. **D** Mature neurons were also positive for synapsin and MAP2. Scale bars represent 50 µm. **E** Neural gene expression of iPSC-derived cortical neurons is similar to postmortem dorsolateral prefrontal cortex (DLPFC) tissues and different from undifferentiated iPSCs. **F** Differential interference contrast (DIC) (left) and fluorescent (right) representative microscopy images taken during electrophysiological recording. **G** Representative whole-cell intracellular recording of spontaneous activity. Inset: two individual action potentials marked by an arrow. **H** Representative firing pattern of a recorded neuron following a prolonged high amplitude depolarization current step.

In this study, we generated neurons from STAR*D patient LCLs to identify biomarkers that distinguish patient response to bupropion treatment. LCLs were reprogrammed to iPSCs, rapidly differentiated to mature prefrontal cortex neurons and treated with an antidepressant for several days. We found significant synaptic differences between neurons derived from bupropion responder and nonresponder neurons, including synaptic connectivity and spine length. Furthermore, using RNA-sequencing we showed that these neurons differ in gene expression patterns and identified several genes, which could be used as biomarkers to predict patient response to the antidepressant bupropion (Fig. 1A).

## Materials and methods

### LCL attainment and culture

Ten MDD patient LCLs from the NIH-supported “STAR*D” study were obtained from NIMH Repository and Genomics Resource, a centralized national biorepository for genetic studies of psychiatric disorders. The STAR*D study (ClinicalTrials.gov Identifier: NCT00021528) focused on nonpsychotic MDD in adults seen in outpatient settings. The primary purpose of this research study was to determine which treatments work best if the first treatment with medication does not produce an acceptable response. The study was supported by NIMH Contract #N01MH90003 to the University of Texas Southwestern Medical Center^21^. All LCLs were derived from MDD patients who did not respond to citalopram on level 1 of the STAR*D study. On level 2, all ten patients were treated with bupropion, to which *n* = 5 responded and *n* = 5 did not (Table S1). Patients were defined as responders if they reached over 50% reduction in their Quick Inventory of Depressive Symptomatology, Self-Report (QIDS-SR) score, and their QIDS score was <7, following the bupropion treatment period.

Cells were thawed and maintained in LCL media composed of RPMI 1640 (Thermo Fisher) supplemented with fetal bovine serum (10%) (Thermo Fisher), 1 mM Glutamax (Thermo Fisher), 50 U/ml penicillin and 50 μg/ml streptomycin (Biological Industries) in T25 cell culture flasks at 37 °C, 5% CO_2_. Cells were observed daily under a light microscope and passaged once culture media became acidic.

### iPSC reprogramming and maintenance

Patient-LCL reprogramming toward iPSCs was carried out using the Cytotune 2.0 Sendai reprogramming kit (Thermo Fisher), according to manufacturer’s instructions. Briefly, 2.5 × 10^5^ cells were resuspended in 1 ml of LCL media containing Sendai virus particles at MOI = 5. Infected cells were then transferred to a single well of a 12-well plate. Following centrifugation at 1000 × *g* for 90 min and 6 h of incubation at 37 °C, fresh LCL media was added to the well. The next day, LCL media was replaced and virus particles were washed away. Two days later, cells were transferred to a human embryonic stem cell (hESC)-qualified Matrigel (Corning) coated six-well plates. In the next 9–12 days, media was gradually changed to ReproTeSR (STEMCELL Technologies) until small stem-cell-like colonies emerged. Once colony confluency reached 25%, media was changed to mTeSR1 (STEMCELL Technologies) and from thereon changed daily. Undifferentiated colonies were passaged using Accutase (Sigma) and maintained in hESC-qualified Matrigel-coated six-well plate or 10 cm^2^ dish. Cells were routinely passaged in 1:6–1:10 ratios once a confluency of 70–80% was reached. Cells were routinely tested negative for mycoplasma (Hylabs).

### Cortical neuron differentiation and antidepressant treatment

Cortical neuron differentiation was carried out as previously described with modifications^23^. iPSCs were counted and seeded at a density of 3 × 10^6^ cells/well on hESC-qualified Matrigel-coated six-well plates and incubated at 37 °C, 5% CO_2_ overnight. Once 100% confluency was reached, differentiation was initiated by changing the media to ectoderm induction media, consisted of knockout DMEM (Thermo Fisher), 15% Knockout serum replacement (Thermo Fisher), 1 mM Glutamax, 100 mM nonessential amino acids (Sigma), 50 mM β-mercaptoethanol (Thermo Fisher), 50 U/ml penicillin and 50 μg/ml streptomycin. In the first 2 days, the media was supplemented with 250 nM LDN193189 (Peprotech), 10 µM SB431542 (Peprotech), and 5 µM XAV939 (Peprotech). In the following 2 days, media was additionally supplemented with 8 µM PD0325901 (Peprotech), 10 µM SU5402 (Peprotech), and 10 µM DAPT (Peprotech). From day 4, media was gradually changed to neuronal differentiation media, consisted of neurobasal media (Thermo Fisher), 1x N2 supplement (Thermo Fisher), 1x B27 supplement (Thermo Fisher), 1 mM Glutamax, 50 U/ml penicillin, and 50 μg/ml streptomycin. On day 6, LDN193189, SB431542, and XAV939 were removed completely. On day 8, neuronal maturation media was also supplemented with 20 ng/ml BDNF (Peprotech), 0.5 mM dibutyryl cAMP (Peprotech), and 0.2 mM ascorbic acid (Sigma). On day 9, differentiated neurons were replated using Accutase and 10 mg/ml DNase I (Sigma) on either glass-bottomed 96-well plates or 24-well plates coated with 15 μg/mL Poly-ornithine (Thermo Fisher), 1 μg/mL Laminin (Sigma), and 2 μg/mL Fibronectin (Sigma). Neurons were spun down and seeded at a density of 150,000 cells/cm^2^. Media was changed 4 h after replating. Media was changed thereafter every other day with neural maturation media including neurobasal media, 1x B27 supplement, 1 mM Glutamax, 0.6% glucose (Sigma), and 50 U/ml penicillin and 50 μg/ml streptomycin, supplemented with 20 ng/ml BDNF, 0.5 mM dibutyryl cAMP, 0.2 mM ascorbic acid, and 10 µM DAPT. From day 15 onward, 1 µg/ml laminin was also added to the media. On day 28 following differentiation, iPSC-derived cortical neurons were treated with either 10 µM bupropion (Tocris Bioscience) or vehicle (PBS, Biological industries) until experiment termination 7 days later.

### Immunofluorescence staining and imaging analysis

Neurons were fixed with 4% paraformaldehyde solution on day 35. Antigen blocking and cell permeabilization were performed using 10% horse serum (Sigma), 0.2% Triton X-10 (Sigma), and 0.5% bovine serum albumin (Sigma) in PBS. Cells were incubated with primary antibodies diluted in blocking solution for 1 hour at room temperature. Fluorophore-coupled secondary antibodies were also diluted in blocking solution and were used for 1 hour at room temperature. All antibodies are listed in Supplementary Table S2. Cultures were counter-stained with 4′,6-diamidino-2-phenylindole (Sigma). Imaging was performed using Nikon Spinning Disk Confocal microscope. For imaging of presynaptic and postsynaptic markers, images were acquired with a ×100 objective in z-stacks.

For dendritic spines imaging, cells were infected on differentiation day 20 with IncuCyte NeuroLight Red Lentivirus—Synapsin Promoter (Sartorius). Cells were fixed at day 35 following 7 days of drug treatment and stained using an antibody against mKate2 expression as described above. Images were acquired using the Nikon Confocal A1R with a ×60 objective and a ×2 digital zoom in z-stacks. Dendritic and spine morphology was assessed using Neurolucida (MBF Bioscience).

Presynaptic and postsynaptic marker analysis was performed using CellProfiler and total dendritic length was measured using Fiji.

### Flow cytometry (FACS) analysis

FACS staining and analysis were carried out using TRA-1-60- and NCAM PE-conjugated antibodies (Miltenyi Biotec) according to manufacturer’s instructions. Cells were sorted and analyzed using Cellstream Flow Cytometer (Merck).

### RNA extraction, sequencing, and analysis

Total RNA was extracted using NucleoSpin RNA XS kit (Macherey-Nagel), according to manufacturer’s instructions. RNA-sequencing libraries were prepared using KAPA Stranded mRNA-Seq Kit with Illumina Truseq adapters according to manufacturer’s instructions and were sequenced using Illumina NextSeq 500 to generate 75 bp single-end reads. RNA-sequencing samples were aligned to the GRCh38 reference genome using STAR aligner^24^. TMM normalization of RNA read counts and differential gene expression analysis were carried out using edgeR^25^. Category enrichment of differentially expressed genes (DEGs) analysis was carried out using the Database for Annotation, Visualization and Integrated Discovery^26^. Accession numbers of obtained data sets are found in Supplementary Table S3. Data were visualized using R-studio.

### Electrophysiology

Electrophysiological recordings were performed as previously described^27^. In brief, prior to recordings, media for iPSC-derived cortical neurons differentiated on glass cover slips was replaced with an artificial cerebrospinal fluid. The recordings were made at 35 °C for up to 2 hours following cell extraction from the incubator. Cells were identified under a DIC microscope (Olympus BX61WI) using a ×20 Olympus objective. Recordings were done using Axoclamp 700B amplifier. Data were visualized using Python, R, and Prism.

### Statistical analysis

Cells from ten MDD patients (*n* = 10) were used in this study, five showing remission following bupropion treatment (*n* = 5) and five that did not (*n* = 5) (Table S1). One iPSC line derived from each group was differentiated twice toward cortical neurons (technical repeats), while the other four were differentiated once (biological repeats). Student’s *t* test were performed one sided and corrected for multiple testing (FDR correction) when required. Investigators were blinded to the remission type of each line until data collection was concluded and before comparisons were carried out.

## Results

### Patient selection from STAR*D cohort

To compare between MDD patients who responded to bupropion and those who were nonresponders, patients were selected from the STAR*D study cohort according to the 16-item QIDS-SR scores^21,22,28^. QIDS-SR scores were compared between the two groups and revealed a significant change in the responder group (*p* < 0.00001) and a nonsignificant change in the nonresponder group (*p* < 0.32) following treatment. Patient age and sex were similar in both groups (Table S1).

### Patient LCLs rapidly give rise to karyotypically normal iPSCs

STAR*D study patient LCLs were briefly expanded before being reprogrammed into iPSCs using non-integrating Sendai virus. After ~3 weeks, pluripotent colonies emerged, exhibiting typical morphology (Supplementary Fig. S1A). iPSC colonies expressed the pluripotency markers OCT4, NANOG, SSEA4, TRA-1-81, and TRA-1-60 and lacked the expression of the differentiation marker SSEA1, consistent with undifferentiated cells (Supplementary Fig. S1B, C). The newly formed cell lines were also shown to be pluripotent, demonstrated by their ability to directly differentiate in vitro into the three embryonic germ layers, ectoderm, endoderm, and mesoderm (Supplementary Fig. S1D). Importantly, iPSC lines were shown to be karyotypically normal (Supplementary Fig. S1E). Together, these results exhibit the ability to rapidly and consistently generate iPSC lines from MDD patient LCLs.

### MDD–iPSCs differentiate to functional cortical neurons

Following the establishment of iPSC lines, cells were rapidly differentiated into cortical neurons (Fig. 1A)^23^. Undifferentiated cells were first differentiated toward neural progenitor cells (NPCs), expressing the characteristic NPC markers PAX6 and Nestin after 5 days in culture (Fig. 1B). Neurons matured over the course of an additional 18 days, expressing the cortical neuronal markers TBR1, BRN2, and MAP2, as well as the synaptic marker synapsin (Fig. 1C, D). RNA-sequencing analysis revealed gene expression similarities between iPSC-derived cortical neurons and postmortem dorsolateral prefrontal cortex tissues (Fig. 1E). An expression divergence of neuronal and pluripotency markers between the differentiated neurons and undifferentiated cells was also detected (Fig. 1E).

Unbiased principal component analysis of global gene expression revealed that these iPSC-derived cortical neurons clustered together with other, previously published MDD patient-derived neurons, and distinctly separated from undifferentiated iPSCs (Supplementary Fig. S1D and Supplementary Table S3). Furthermore, iPSC-derived cells also cluster together with fetal-brain tissues, derived from embryos 13–18 weeks postconception (Supplementary Fig. S1F and Supplementary Table S3). All iPSC-derived neurons clustered separately from postmortem dorsolateral prefrontal cortex anterior cingulate cortex and nucleus accumbens tissues, highlighting persistent differences between in vitro and fetal cultures and postmortem tissues (Supplementary Fig. S1F and Supplementary Table S3). Category enrichment analysis of the top 400 genes upregulated following differentiation, revealed a significant enrichment of relevant categories including neurogenesis (FDR < 7.2E^−13^) and synapse (FDR < 1.3E^−6^) (Supplementary Fig. S1G). Enrichment of neurogenesis could imply that, similarly to other iPSC-derived neurons and as expected, these neurons still resemble a fetal brain.

In order to estimate the similarities and differences between the two remission groups and their heterogeneity, we evaluated the expression of the 45 genes that comprise the GO annotation term “neuron maturation” (GO:0042551) (Supplementary Fig. S2). While some expected heterogeneity between cultures appeared, the vast majority of genes were similar between samples and between responders and nonresponders. Importantly, the difference between the groups was not significant for any of these genes following FDR correction for multiple comparisons (*p* > 0.05).

To test the functionality of the generated neurons, we performed whole-cell intracellular recordings of 24 individual neurons from two cell lines, originated from four cultures (Fig. 1F–H and Supplementary Fig. S1F, G). Action potentials were observed in two-thirds of the cells, either spontaneous (Fig. 1G) or evoked (Fig. 1H). In agreement with previous studies^27,29^, measured passive membrane properties varied between neurons (Supplementary Fig. S1H). Resting membrane potential was −41 ± 13 mV (mean ± SD), membrane resistance was 1.1 ± 1.0 GΩ, and membrane time constant was 18 ± 12 ms (Supplementary Fig. S1H). Action potential amplitudes were in the expected range, between 20 and 60 mV (39 ± 13 mV), with a duration slightly longer than expected (7 ± 5 ms). Threshold potential was in an expected range, −33 ± 4 mV (Supplementary Fig. S1G).

Together, these data suggest that MDD patient iPSC-derived cortical neurons express neuronal genes and proteins and are electrically functional, thus they can be used as a relevant disease model for neuronal-based diseases.

### Responder-derived neurons exhibit synaptic changes following bupropion treatment

**Fig. 2:**
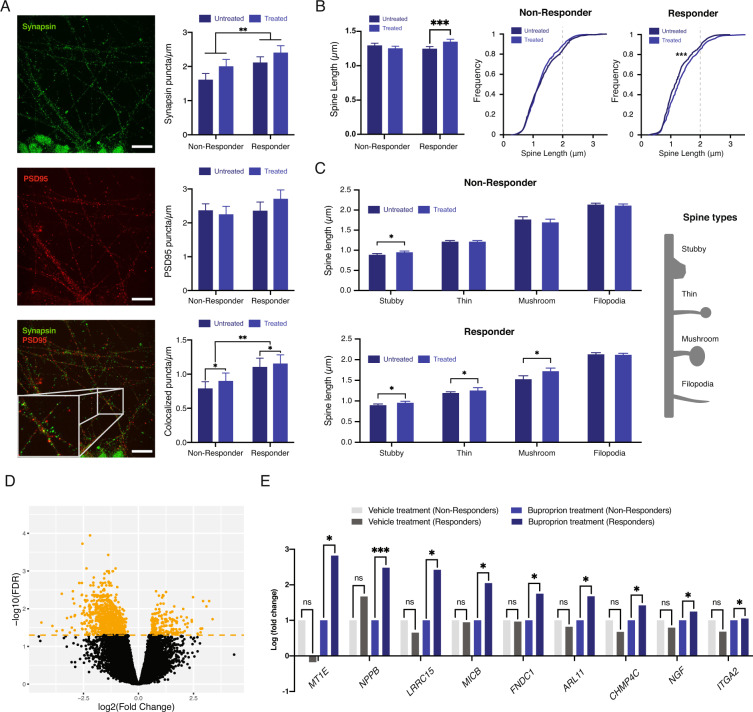
iPSC-derived cortical neurons mimic patient response to an antidepressant. **A** Immunofluorescence images and quantifications of the presynaptic marker Synapsin (top panel), postsynaptic marker PSD95 (middle panel), and colocalized markers (bottom panel) in nonresponder untreated (*n* = 5 patients, *n* = 52 dendrites), nonresponder bupropion treated (*n* = 5 patients, *n* = 54 dendrites), and responder patient iPSC-derived cortical neurons untreated (*n* = 5 patients, *n* = 39 dendrites) and treated with bupropion (*n* = 5 patients, *n* = 47 dendrites). Scale bars represent 10 µm. **B** Average dendritic spine length was significantly altered following bupropion treatment only in patient responder-derived cortical neurons, both in average length and in spine length frequency. **C** Average dendritic spine length in specific spine types. Spine length was significantly changed in patient responder-derived neurons in three out of four spine types. Spine types are illustrated (right panel). **D** Volcano plot of statistically significant, differentially expressed genes in responder and nonresponder patient-derived cortical neurons following bupropion treatment. Orange line represents FDR < 0.05 (N = 10, *n* = 5 for each remission group). **E** Identified biomarker gene expression significantly changed in responder patient-derived cortical neurons following bupropion treatment. One-sided Student’s *t* test was used for comparisons. *p < 0.05, **p < 0.01, ***p < 0.001. Error bars represent standard error of the mean.

Synaptic changes are well established hallmarks in the brains of MDD patients^5,6,9^. Following the establishment of a robust reprogramming and differentiation platform, we sought to identify morphological synaptic biomarkers able to distinguish between responders and nonresponders to bupropion treatment. Differentiated neurons expressed the presynaptic marker synapsin and the postsynaptic marker PSD95, allowing for synapse-colocalization analysis (Figs. 2A and S3A). The number of synapsin puncta was significantly elevated in neurons derived from bupropion responders compared with nonresponders, both following vehicle and bupropion treatment (2.14 ± 0.14 vs. 1.64 ± 0.16 and 2.43 ± 0.18 vs. 2.03 ± 0.18 puncta/μm, respectively, mean ± SEM, *p* < 0.01) (Fig. 2A). Although the cohorts did not significantly differ in the number of PSD95 puncta, responder-derived cortical neurons had significantly more colocalized synapses in comparison with nonresponder cells, both at basal levels and following treatment (1.14 ± 0.12 vs. 0.81 ± 0.08 and 1.31 ± 0.14 vs. 0.91 ± 0.11 colocalized puncta/μm, respectively, mean ± SEM, *p* < 0.01). Of note, nonresponder neurons also showed an elevation in colocalized synapses following exposure to bupropion, but it was significantly lower compared to responder-derived cells (Fig. 2A).

### Bupropion treatment alters dendritic spine morphology in responder cells

Dendritic spine morphology is a well-characterized indicator of spine maturation, renewal, and loss^30^, potentially rendering it a promising biomarker for antidepressant response. Dendritic spine morphology of patient-derived cortical neurons was therefore evaluated following bupropion treatment using three measurements: total spine length, distribution of spine length, and lengths of specific spine types (Fig. S3B).

We found that responder-derived neurons exhibited significantly longer spines following antidepressant exposure (1.26 ± 0.02 µm vs. 1.37 ± 0.02 µm, mean ± SEM, *p* < 0.001), while neurons from nonresponders showed a small, nonsignificant reduction in spine length (Fig. 2B). A closer look at the cumulative distribution of spine length revealed that while nonresponder-derived cells had a similar distribution following bupropion treatment compared to vehicle-treated controls, responder-derived cortical neurons exhibited a significantly different distribution following antidepressant exposure, with the greatest difference demonstrating being an increase in medium-sized synapses (1–2 µm) (*p* < 0.001) (Fig. 2B).

The alterations in medium-sized synapse frequency led us to further look into the different spine types following bupropion or vehicle treatment. Dendritic spines are commonly categorized according to their shape^31^. We focused on the length of four spine types: stubby, thin, mushroom, and filopodia, which differ in shape and size (Fig. 2C). Nonresponder-derived cortical neurons exhibited similar lengths of different spine types, except for a significant increase in that of stubby synapses (0.91 ± 0.02 vs. 0.97 ± 0.02 µm following treatment, mean ± SEM, *p* < 0.05). In contrast, responder-derived cortical neurons showed a significant increase in the length of stubby (0.91 ± 0.02 vs. 0.97 ± 0.02 µm, mean ± SEM, *p* < 0.05), thin (1.21 ± 0.02 vs. 1.27 ± 0.02 µm, mean ± SEM, *p* < 0.05) and mushroom synapses (1.54 ± 0.07 vs. 1.74 ± 0.06 µm, mean ± SEM, *p* < 0.05), and maintained a similar length of filopodia-type synapses. Together, responder-derived neurons exhibited overall longer spines, a significantly different spine length distribution, and an increase in specific spine-type length, demonstrating a multi-faceted synaptic response bupropion treatment in vitro. This significant difference between responder and nonresponder-derived neurons following drug treatment may serve as a biomarker to predict patient responses to bupropion treatment.

### Gene expression changes following antidepressant treatment

Global gene expression changes following bupropion treatment, assessed by RNA-sequencing, showed that the expression of numerous genes was significantly altered between responder and nonresponder neurons following treatment (Fig. 2D). Category enrichment analysis of the DEGs posttreatment revealed a significant enrichment of relevant categories including psychiatric diseases, neurons projection, and neurological diseases (Fig. S3C), suggesting that the gene expression differences observed in our model resemble changes previously observed in patients in vivo.

Additional DEG analysis revealed that many of the expression differences identified between responder- and nonresponder-derived neurons were also significantly different in their expression prior to bupropion treatment. In order to identify genes that could serve as response markers following treatment, we identified genes that were significantly upregulated following treatment in responder neurons only (*p* < 0.05) (Fig. 2E). This group of genes included Metallothionein 1E (*MT1E*), Natriuretic Peptide B (*NPPB*), Leucine Rich Repeat Containing 15 (*LRRC15*), MHC Class I Polypeptide-Related Sequence B (*MICB*), Fibronectin Type III Domain Containing 1 (*FNDC1*), DP Ribosylation Factor Like GTPase 11 (*ARL11*), Charged Multivesicular Body Protein 4C (*CHMP4C*), Nerve Growth Factor (*NGF*), and Integrin Subunit Alpha 2 (*ITGA2*) many of which have already been implicated in MDD and could be used to identify patient response to bupropion in vitro, prior to drug prescription.

## Discussion

MDD affects millions of people around the world and is a leading global cause of morbidity^2^. Despite the increasing availability of antidepressants, the inability to predict their effect in patients remains a substantial hurdle, resulting in successful outcomes for merely two-thirds of MDD patients after weeks to months of treatment^4^. A rapid identification of the medication that will benefit each patient could ameliorate symptoms faster, potentially reducing high suicide prevalence in MDD patients and improving patient lives. In contrast to animal models, patient cells are an appealing platform to achieve this goal, offering the opportunity for a rapid, patient-personalized, screen of drug efficacy and a readout of neuronal specific biomarkers.

We have utilized patient-derived LCLs to generate cortical neurons. Patient cell reprogramming is predominantly initiated from fibroblasts, although numerous cell types have been suggested to replace them^32^. While LCLs give rise to iPSCs similar to those derived from fibroblasts^33,34^, the latter have been shown to carry numerous UV radiation-related mutations and genetic alterations, potentially influencing the phenotypes observed in iPSCs^19,35^. Fibroblast derivation also requires an invasive skin biopsy, a substantial hurdle in their use. In contrast, multiple repositories worldwide contain LCLs with patient clinical history, though these are widely ignored in disease modeling research and the iPSC field^34^. This is surprising, as LCLs, together with other cell types such as peripheral blood mononuclear cells, can be easily obtained from patients, promoting personalized medicine and treatment. Using human samples collected as part of the STAR*D study we showed that LCLs are a reliable source for patient cell reprogramming and cortical neurons generation thereafter. A potential drawback from a broad use of LCLs could be concerns about genetic and epigenetic changes following EBV immortalization. However, a recent report showed that single nucleotide polymorphisms are rather stable following transformation^36^. Epigenetic changes in LCLs have also been identified, although they continue to share many modifications with their cells of origin^37,38^. Nevertheless, these concerns should be addressed when using LCLs for iPSC reprogramming.

Antidepressants have substantial side effects which vary between individuals^12^. SSRIs are generally used as a first line of treatment for MDD patients, as they have fewer side effects compared with earlier-generation antidepressants^12^. Upon treatment failure, physicians typically prescribe a norepinephrine–dopamine reuptake inhibitor, such as bupropion^39^. Alongside exhibiting similar antidepressant effects as SSRIs, bupropion has been shown to have fewer side effects^39,40^, making it a popular alternative.

Several neural biomarkers have been suggested to predict antidepressant response in humans, including brain activity in response to conflict^41^, intrinsic brain network activity^42^, and intrinsic differences in serotonergic neuron morphology^11^. However, none of those seem to apply to all MDD patients^42^. In this work, we provide several biomarkers that can be used in vitro to predict bupropion effects in MDD patients, using patient cells, readily transformed from blood, acquired from males and females from a diverse age group.

We found that following bupropion treatment, cortical neurons derived from cells of patients that responded to bupropion treatment had significantly different synaptic morphology compared with vehicle treatment. These features included enhanced colocalization of presynaptic and postsynaptic markers, overall longer spines and an altered abundance of spine subtypes. The enhanced colocalization of synaptic markers observed in responder-derived cells could be indicative of enhanced connectivity following antidepressant treatment. MDD patients have been shown shown to present reduced global functional connectivity in comparison with healthy individuals^43,44^. Bupropion treatment has been suggested to increase functional connectivity^45^, a feature that the responder-derived cortical neurons recapitulate.

In contrast to stubby spine length, which was elevated in both responder and nonresponder-derived cells following antidepressant treatment, thin and mushroom spines were longer only in responder-derived neurons. In contrast to the immature stubby and filopodia spines, thin and mushroom spines represent more stable structures, previously referred to as “learning” and “memory” spines, respectively^46^. The robust, significant increase in their length in responder-derived neurons following antidepressant treatment suggests that bupropion responsiveness could include synaptic maturation. Together, these synaptic changes could be used as a biomarker for bupropion effects in vitro. It is noteworthy that microscopy-based high content screening has been shown to be feasible to detect synaptic changes^47^, further highlighting the potential use of our results.

Gene expression changes could also serve as a valuable biomarker for prediction of antidepressant responsiveness, as they can be easily and routinely monitored and have been previously linked to MDD^10^. Using an unbiased RNA-sequencing analysis we were able to identify nine genes that were significantly upregulated in responder-derived neurons. This is the first time that five of these genes, including *NPPB, LRRC15, MICB, CHMP4C, ITGA2*, are found to be involved in MDD changes. Encouragingly, the other four were previously suggested to be altered in MDD models. *MT1E* was found to be downregulated in brains of MDD patients who committed suicide^48^, suggesting its elevation in bupropion responders following treatment could be indicative of symptom amelioration. *FNDC1* was associated with mood and therapeutic effects in a rodent depression model^49^ and *ARL11* was differentially expressed in blood of depressed patients^50^. *NGF* was previously suggested to be a depression biomarker, as its serum levels differ between MDD patients and a control group^51^. However, results regarding its levels in control and depressed patients are debatable and conflicting^52^. Although these previous findings are more correlative than causative, transcript abundance of the same genes in our model agrees with these reports and suggests the expression level of these nine genes could also be used as a biomarker for depression-related symptoms in cortical neurons in vitro. Interestingly, significant gene expression differences between responder- and nonresponder-derived neurons were also observed at baseline. It remains undetermined if these changes could also serve as biomarker for treatment response, without in vitro drug treatment.

It is important to mention several limitations of our platform. While a 7-day treatment with bupropion was sufficient to generate significant and relevant phenotypes, it is shorter than the weeks required for its function in vivo. It also remains to be seen if the biomarkers identified are relevant for other antidepressants, or specific to bupropion. Furthermore, a larger cohort of patients is required to further establish them as a fixed set to determine their ability to predict, retrospectively as well as prospectively, the ability of bupropion to ameliorate depression symptoms in patients.

As the prevalence of MDD and the variety of antidepressants grow, it is important to move away from the “trial-and-error” approach physicians use when choosing a treatment for their patients. Our study utilized patient-derived cortical neurons to identify biomarkers relevant for bupropion responsiveness. Identifying and using such biomarkers in vitro can potentially revolutionize the way we treat MDD, enhancing symptom remission by treating each patient with the most suitable medication for them, preventing further suffering and loss of life.

## Supplementary information

Supplemental Figure Legends

Supplemental Figure S1

Supplemental Figure S2

Supplemental Figure S3

Supplemental Table S1

Supplemental Table S2

Supplemental Table S3
