# Supplementary material for: Depression patient-derived cortical neurons reveal potential biomarkers for antidepressant response
Source: Transl Psychiatry. 2021 Apr 1;11:201. doi: 10.1038/s41398-021-01319-5 (PMC8016835; doi:10.1038/s41398-021-01319-5)
Supplement: Supplementary file 1 — Supplemental Figure Legends [file 41398_2021_1319_MOESM1_ESM.docx]

**Supplemental figure legends**

**Figure S1. iPSCs reprogrammed from MDD patient LCL lines show normal karyotype and colony morphology, and iPSC-derived neurons are comparable to fetal brain and other differentiation protocols with functional characteristics.** (A) Phase images of LCLs (left) and undifferentiated iPSC colony (right), (B) iPSC colonies were positive for the undifferentiated cell marker TRA-1-60 as validated by flow cytometry, (C) iPSC colonies were positive for undifferentiated cell markers as shown by immunofluorescence of OCT4, NANOG, TRA-1-81, SSEA4 and TRA-1-60 and negative for the differentiation marker SSEA1. (D) iPSC pluripotent capacity was validated using a trilineage differentiation protocol and subsequent immunofluorescent staining for PAX6 (ectodermal marker), SOX17 (endodermal marker) and brachyury (mesodermal marker). (E) Karyotype spreads of two iPSC lines show normal 46+XX karyotype. (F) global gene expression of iPSC-derived cortical neurons (pink) cluster together with iPSC-derived cortical neurons from other published papers (reds) and with fetal brain tissues from 13-16 weeks postconception (gray), distinct from undifferentiated iPSCs (black) and from postmortem (pm) brain tissues (browns). (G) Category enrichment analysis of the 400-top genes upregulated following differentiation of iPSC towards cortical neurons obtained using DAVID^26^. (H) Passive electrophysiological properties of iPSC-derived neurons included resting membrane potential (V_rest_), membrane input resistance (R_m_) and membrane time constant (τ_m_) of the recorded cells. (N=24, 12 from each of two cell lines from four technical replicates of each cell line). (I) Active electrophysiological properties included threshold potential (V_th_), action potential (AP) amplitude and duration (N=16, 7 from one cell line and 9 from a second one, from four technical replicates of each cell line).

**Figure S2. Heterogeneity in neuronal maturation between and within remission groups.** Gene expression for the 45 genes comprising the GO term “neuron maturation” was evaluated in five bupropion responder cultures and six bupropion non-responder cultures using RNA-sequencing. Gene expression differences between groups were statistically insignificant for all genes (Students’ T-test p>0.05).

**Figure S3. Phenotype clarification of neurons derived from patients with depression.** (A) Schematic illustration of the colocalized signal obtained from pre- and postsynaptic markers. (B) Dendritic spine morphology of iPSC-derived cortical neurons visualized using lentiviral-based neuronal labeling driven by a human Synapsin promoter (LV-Synapsin-mKate2). Scale represents 20 µm. (C) Category enrichment of differentially expressed genes following bupropion treatment between responders and non-responders, obtained using DAVID^26^.

**Table S1. Summary of MDD patient cell line demographics used in the study.** QIDS: Quick Inventory of Depressive Symptomatology; HRSD: Hamilton Depression Rating Scale.

**Table S2. List of antibodies used in the study.**

**Table S3. List of accession numbers used for RNA sequencing comparison.** All accession numbers appear on the National Center for Biotechnology Information (NCBI) Sequence Read Archive (SRA) alongside the PubMed ID (PMID) of their original paper.
