# Supplementary figures and images for: Depression patient-derived cortical neurons reveal potential biomarkers for antidepressant response
Source: Transl Psychiatry. 2021 Apr 1;11:201. doi: 10.1038/s41398-021-01319-5 (PMC8016835; doi:10.1038/s41398-021-01319-5)

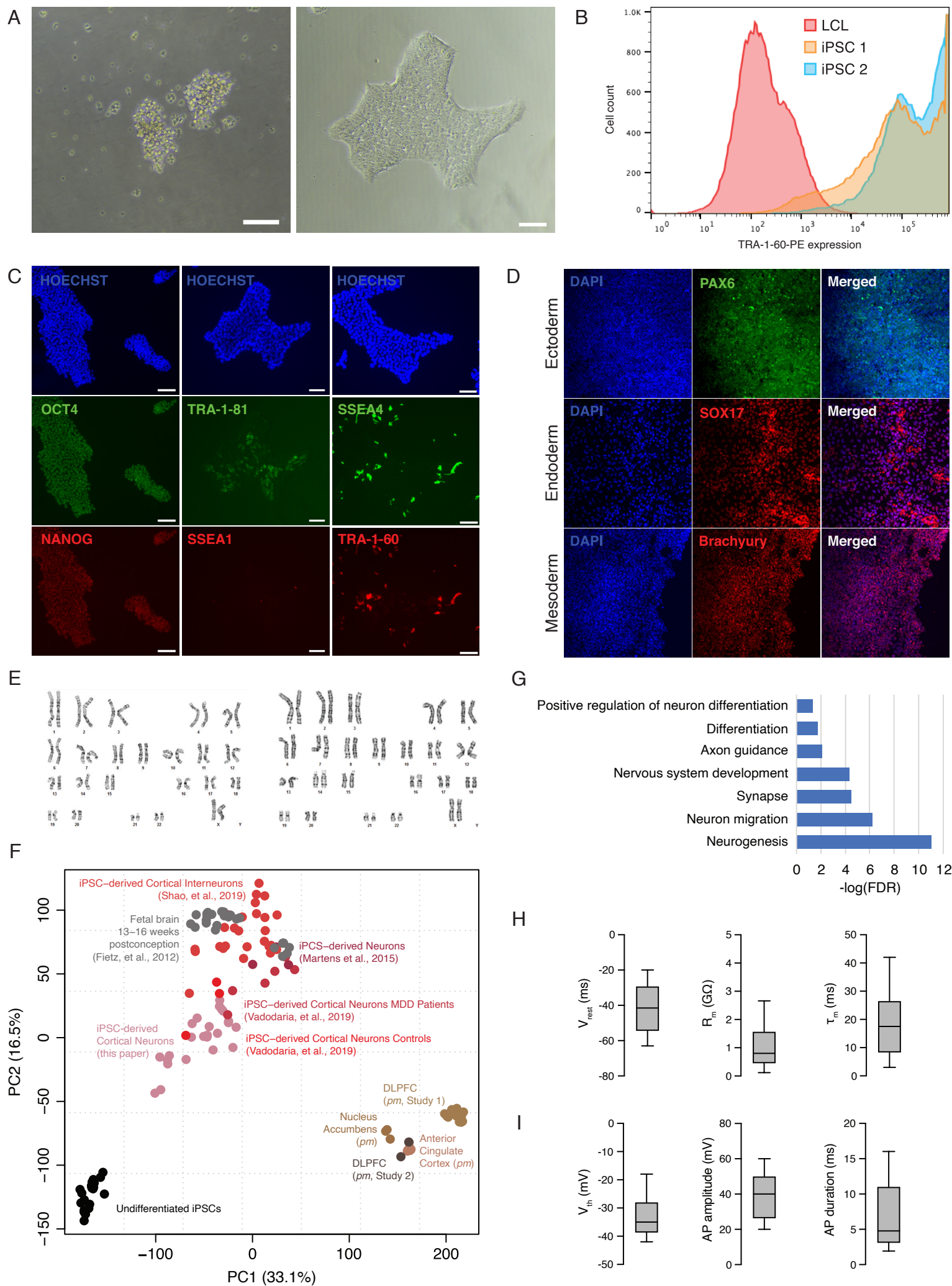

Supplement: Supplementary file 2 — Supplemental Figure S1 [file 41398_2021_1319_MOESM2_ESM.pdf]

## Neuronal Maturation GO term Genes (GO:0042551)

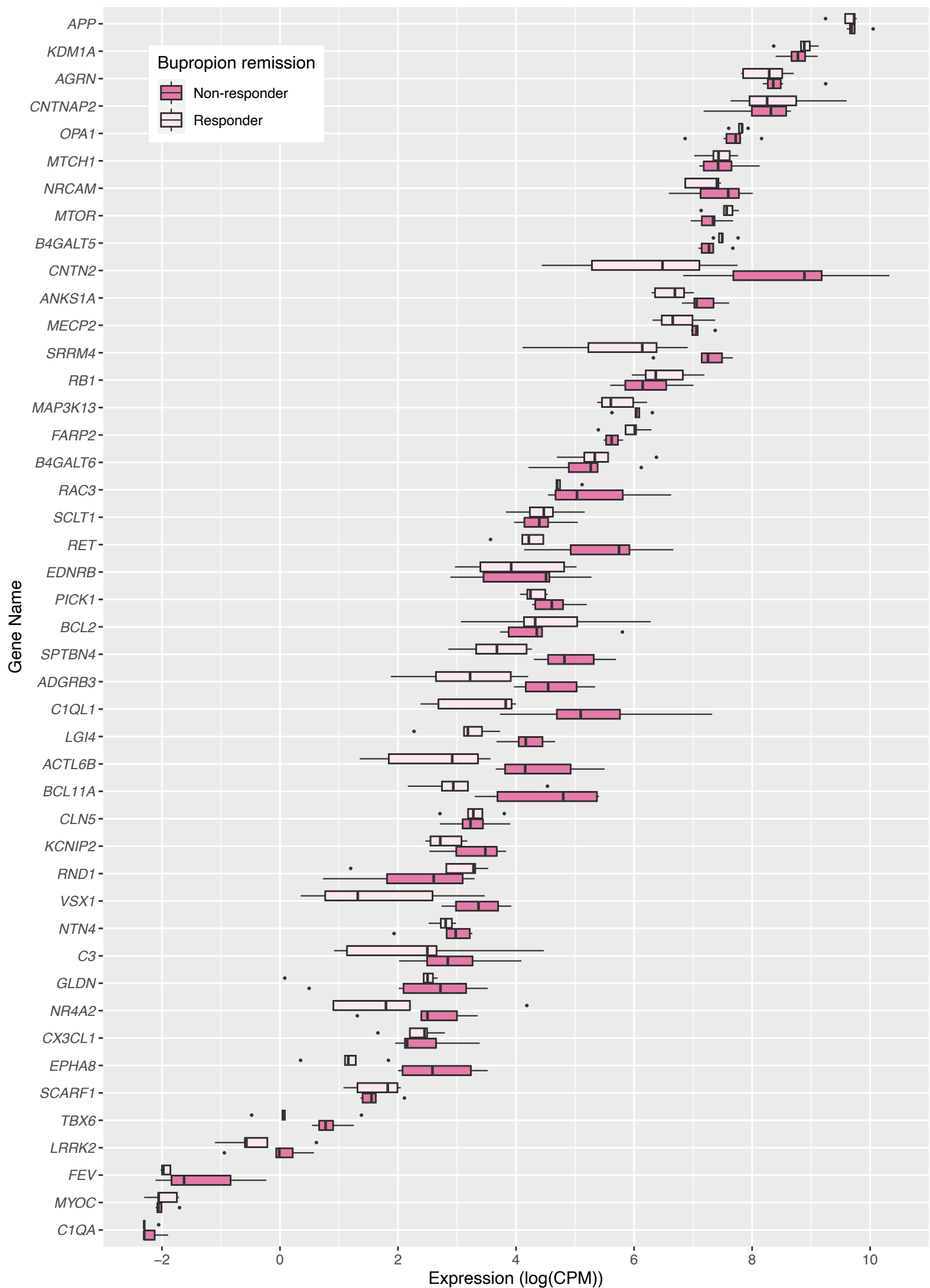

Supplement: Supplementary file 3 — Supplemental Figure S2 [file 41398_2021_1319_MOESM3_ESM.pdf]

A

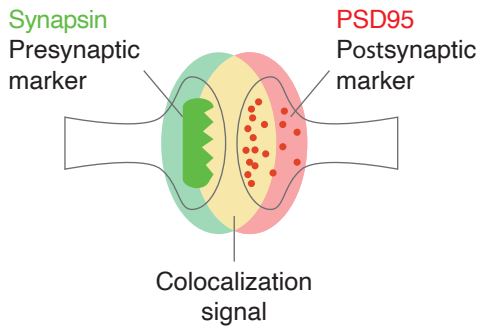

B

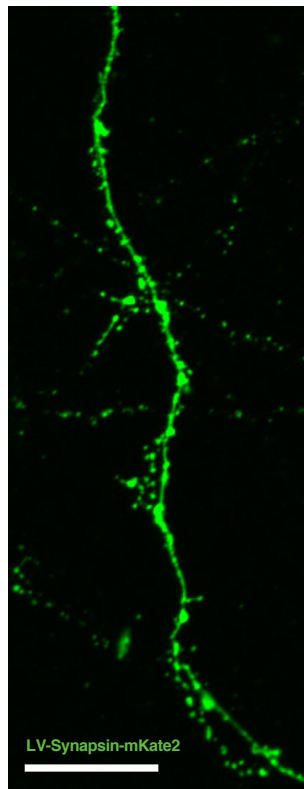

C

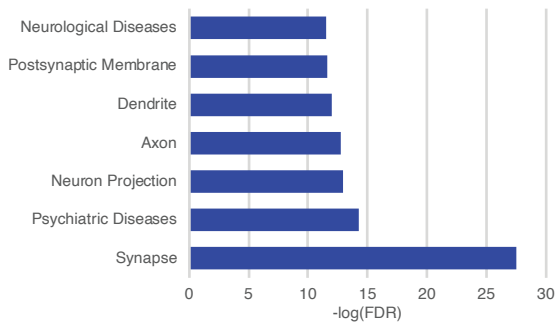

Supplement: Supplementary file 4 — Supplemental Figure S3 [file 41398_2021_1319_MOESM4_ESM.pdf]
